# P-656. Investigating the Current Status and Exploring Issues for the Construction of Ventilator-Associated Pneumonia Surveillance

**DOI:** 10.1093/ofid/ofae631.853

**Published:** 2025-01-29

**Authors:** Osamu Kanai, Takanori Ito, Kohei Fujita, Kiminobu Tanizawa

**Affiliations:** National Hospital Organization Kyoto Medical Center, Kyoto, Kyoto, Japan; Kyoto Medical Center, Kyoto, Kyoto, Japan; National Hospital Organization Kyoto Medical Center, Kyoto, Kyoto, Japan; Kyoto Medical Center, Kyoto, Kyoto, Japan

## Abstract

**Background:**

Ventilator-associated pneumonia (VAP) is a major infectious complication in invasive mechanical ventilation (IMV)-controlled patients. Because VAP adversely affects multiple outcomes, including risk of death, length of intensive care unit (ICU) stays, and length of hospital stay, appropriate preventive measures need to be constructed. To establish preventive measures for VAP in our hospital, we surveyed the occurrence of VAP and summarized the issues identified in the process.

**Methods:**

Patients who received 48 hours or more of IMV at our hospital in 2023 were included in this study. We retrospectively studied the number of ventilator-associated conditions (VAC), infection-related ventilator-associated complications (IVAC), and probable/possible VAP (PVAP) events using the ventilator-associated events (VAE) algorithm from the Centers for Disease Control and Prevention National Healthcare Safety Network. We used the records of ventilator settings used by medical engineers (ME) for daily ventilator check-ups to screen for VAC events. Clinical findings, including the results of bacteriological tests of lower respiratory tract specimens, were investigated using electronic medical records.

**Results:**

Of the 143 patients (157 IMV events) who underwent IMV during the study period, 102 (113 IMV events) were eligible patients. There were 23, 8, and 3 events of VAC, IVAC, and PVAP, respectively. The incidence of VAC, IVAC, and PVAP per 100 IMV events was 20.4, 7.1, and 2.7, respectively. The main causes of VAC events other than IVAC and PVAP were pulmonary edema in 4 events and septic shock in 3 events. During this study, screening for VAC events was readily available in the ME inspection records. In contrast, because of the overlap in ordering methods for culture of lower respiratory tract specimens under ventilatory management and self-discharged sputum, it became necessary to comprehensively review the electronic medical record of the patient in question for bacteriological testing.

**Conclusion:**

In our VAP surveillance, the proportion of IVAC and PVAP events in VAC events was small. To ensure smooth VAP surveillance, the method of ordering bacteriological tests needs to be revised.

**Disclosures:**

**All Authors**: No reported disclosures

